# VITAMIN D – A KEY FACTOR IN THE TREATMENT OF ASD PEOPLE?

**DOI:** 10.1192/j.eurpsy.2023.1950

**Published:** 2023-07-19

**Authors:** M. A. Robea, M. I. Balmus, M. Nicoara, A. Ciobica

**Affiliations:** 1 Biology, Doctoral School of Biology, Faculty of Biology, “Alexandru Ioan Cuza” University of Iasi; 2 Exact Sciences and Natural Sciences, Institute of Interdisciplinary Research,”Alexandru Ioan Cuza” University of Iasi; 3 Biology, Faculty of Biology, “Alexandru Ioan Cuza” University of Iasi, Iasi, Romania

## Abstract

**Introduction:**

Autism spectrum disorder (ASD) is a disorder with social, communication and behavioral disturbances that start from early childhood. There are many difficulties in diagnosing people with ASD. The diagnostic criteria are in terms of behavior descriptions, and as methods of intervention the most used is the applied behavior analysis (ABA). Although, the treatment of autism is not based on drugs, there are a number of reports that sustains the vitamin supplementation. For example, the deficiency of vitamin D (VD) was often outlined in the serum of the ASD people. Nowadays, zebrafish (*Danio rerio*) plays an important role in the modeling era; being one of the main organisms used in animal studies.

**Objectives:**

In this study, we aimed to describe the influence of VD in autistic people, and the possibility of vitamin investigation through animal models studies.

**Methods:**

For analyzing this subject specific scientific databases were screened using certain keywords as: ”autism spectrum disorder”, ”vitamin D”, ”treatment”, ”deficiency”,”animal models” and ”zebrafish”. Inclusion criteria were studies that (1) investigated a behavioral intervention, (2) used animal models for ASD modelling, (3) reported vitamin D results, and (4) were published within the last 20 years.

**Results:**

The majority of the studies supported the importance of an adequate level of VD in the body, mainly due to its implication during pregnancy and early brain development. The few existing data bring information about the positive impact of its administration in ASD children; in which a considerable improvement in typical symptoms was observed. For further knowledge about VD activity in ASD it was suggested the animal modelling, especially zebrafish organisms due to its numerous advantages (high similarity of its genome with the human one).

**Conclusions:**

VD deficiency during pregnancy and early brain development is a real risk factor besides genetic predisposition. Moreover, the use of animal models for investigating the effect of VD is required for a better understanding of the vitamin mechanism in ASD people.

Acknowledgement: *R. M.-A. and B. M.-I. are supported by the Project POCU/993/6/13/153322 ”Suport educațional și formativ pentru doctoranzi și tineri cercetători în pregătirea inserției în piața muncii” of the European Social Fund through the Human Capital Operational Program.

**Disclosure of Interest:**

M. Robea Grant / Research support from: Project POCU/993/6/13/153322 ”Suport educațional și formativ pentru doctoranzi și tineri cercetători în pregătirea inserției în piața muncii” of the European Social Fund through the Human Capital Operational Program., M. Balmus Grant / Research support from: Project POCU/993/6/13/153322 ”Suport educațional și formativ pentru doctoranzi și tineri cercetători în pregătirea inserției în piața muncii” of the European Social Fund through the Human Capital Operational Program, M. Nicoara: None Declared, A. Ciobica: None Declared

